# The Spontaneous Resolution of Trauma-Induced Uterine Arteriovenous Malformation

**DOI:** 10.7759/cureus.52098

**Published:** 2024-01-11

**Authors:** Laurence Stolzenberg, Alixandra Ryan, Paul J Hannon

**Affiliations:** 1 Interventional Radiology, Alabama College of Osteopathic Medicine, Dothan, USA; 2 Internal Medicine, Thomas Hospital, Fairhope, USA; 3 Interventional Radiology, Mobile Infirmary Medical Center, Mobile, USA

**Keywords:** radiology, dilation and curettage, interventional radiology, arterial ablation, uterine avm

## Abstract

This paper will describe the case of a woman who presented with a rare condition called uterine arteriovenous malformation (AVM). A uterine AVM represents a connection between veins and arteries in the uterus. Clinicians should always consider this condition for a woman of childbearing age who presents with unexplained vaginal bleeding. In this particular case, a woman had an AVM diagnosed two months following a miscarriage, and a dilation and curettage (D&C), for retained products of conception. This patient presented to the emergency department in apparent distress, although physical examination and initial laboratory values revealed no hemodynamic instability. Ultrasonographic study, followed by an MRI, confirmed the presence of a uterine AVM. Following a consultation with obstetrics and gynecology, she was ultimately referred to an interventional radiologist for a minimally invasive uterine artery embolization. On the day of the procedure, following vascular access, angiography revealed the AVM had spontaneously self-resolved in the interval. In this paper, we will further discuss the possible utility of a repeat ultrasonographic study to reconfirm AVM prior to any surgical procedure, as well as discuss some confounding factors including the use of medroxyprogesterone acetate injections for birth control prior to the formation of the AVM.

## Introduction

An arteriovenous malformation (AVM) is an abnormal and non-functional connection between arteries and veins [1,2]. A uterine AVM is an abnormal connection between arteries and veins that is specifically located in the uterus and involves the uterine arteries [3,4]. These malformations often present with significant hemorrhage, which is usually described as sporadic, although some cases report incidental findings in an asymptomatic patient [1,5]. Additionally, many uterine AVMs present after uterine trauma, ranging from a normal vaginal birth to a dilation and curettage (D&C), as well as numerous other similar causes that are described in the literature [1,5]. Other presenting symptoms of this condition include lower abdominal pain, dyspareunia, and anemia [1,5]. Some rare cases have been described where severe or numerous AVMs have led to volume-depleted cardiovascular repercussions with accompanying symptoms such as dyspnea, fatigue, and cardiac decompensation [5].

According to current literature, diagnosing uterine AVMs still poses a challenge and there is no definite consensus on a specific diagnostic algorithm [6]. Most literature agrees that the first step in diagnosis is a thorough patient history to elucidate possible causes of acquired AVMs, such as recent pregnancy or D&C [1,6,7]. This is usually followed by an ultrasonographic examination, often transabdominal or transvaginal, depending on the preference of the performing gynecologist or radiologist [1,6,7]. It is also stressed by one particular literature review that further diagnostic modalities are strongly recommended, as women have occasionally had concurrent findings relevant to retained products of conception requiring D&C. This procedure could lead to life-threatening bleeding if conducted in the presence of an AVM [6]. Further diagnostic modalities often used include MRI or angiography, both with high specificity and sensitivity [6].

Uterine AVMs are described by most relevant publications as being very rare, with most papers stating there are less than 100 cases available [5,7]. One paper mentions that the reported incidence is on the rise due to the higher availability of advanced imaging and diagnostic methods, although this was not further developed in the paper in question, and not supported by other papers [5]. Further, a large literature review described 39 women with ultrasonographic findings suspicious of AVMs. However, further diagnostic modalities revealed different pathophysiological processes, confirming the absence of AVMs in all but five of the patients in the review mentioned above demonstrating the importance of further imaging modalities for diagnostic confirmation [6].

The etiology of uterine AVMs is usually classified into two categories. The first, congenitally acquired AVMs, are reported as the least common type, often occurring in women of reproductive age, and very rarely seen in women who have never been pregnant [5,8]. Acquired AVMs are typically related to uterine trauma, further characterized as iatrogenic or non-iatrogenic [5,9]. The often-cited iatrogenic causes include D&C or uterine surgery, such as cesarean section (C-section) [5,9]. Non-iatrogenic causes include vaginal delivery and rarer pathologies such as trophoblastic disease and various uterine neoplasms [5,9].

Prognosis and treatment vary widely based on hemodynamic stability upon presentation and history of heavy bleeding, as well as the patient’s desire for future fertility [1]. In cases of acute bleeding or hemodynamic instability, the literature describes both medical management and blood transfusion as required, as well as surgical options, including embolization or even hysterectomy [1,5]. Medical management described includes multiple modalities to reduce uterine bleeding, including high-dose progesterone or methylergonovine [1]. When there is no acute hemodynamic instability, and no history of heavy bleeding, expectant management may be warranted with close follow-up [1]. A case review identifying multiple patients with uterine AVMs described varying numbers of spontaneous resolutions, with their conclusion demonstrating resolution without further management in six out of nine cases [10].

## Case presentation

This patient was a 24-year-old female who presented to the emergency department with a chief complaint of sudden-onset heavy vaginal bleeding. She mentions that the onset of bleeding was approximately four hours prior to her arrival and was accompanied by abdominal cramping. She describes the onset as sudden and without provocation. She attempted to contain the bleeding with a pad that was quickly saturated and contained clots. She mentioned it had been somewhat intermittent over the last four hours, with the initial episode ceasing spontaneously, but she began bleeding again “a few” times since. She denied dysuria, flank pain, or any bowel or bladder complaints.

The patient denied any significant medical history and described herself as being healthy. She suffered a miscarriage approximately two months prior that required D&C for retained products of conception, and she was subsequently placed on medroxyprogesterone. She denied any menstrual period since the miscarriage, although she endorsed having regular periods with no abnormal bleeding before pregnancy. The patient denied smoking or using smokeless tobacco products, endorsed approximately one standard drink of alcohol per week, and denied any current or recent illicit drug use.

Vital signs in the emergency department revealed an elevated blood pressure of 141 millimeters of mercury (mmHg)/88 mmHg, a pulse of 93 beats per minute (BPM), a temperature of 98.6 degrees Fahrenheit (°F), a respiratory rate of 15 breaths per minute and a blood oxygen saturation of 99% on finger pulse oximetry. A physical examination, conducted with a chaperone, revealed a normal cardiovascular exam, no signs of volume depletion, and normal respiratory and gastrointestinal examination. Further genitourinary examination revealed a normal vulva with no active bleeding, but remnants of bleeding in the external cervical os without adnexal tenderness. The patient equally denied costovertebral angle (CVA) tenderness. The patient was given IV fluids, and a set of standard laboratory tests were ordered. Initial laboratory investigations revealed the values included below (Table 1).

**Table 1:** Initial laboratory investigations

Laboratory Results	Measured Value	Expected/Standard Values
Hemoglobin	13.7 g/dL	Female: 12.1 to 15.1 g/dL
Hematocrit	40.2%	Female: 36 to 48%
Mean Corpuscular Volume (MCV)	88.5 fL	80-100 fL
White Blood Cell Count (WBC)	7,100 cells/uL	4500-11,000 cells/uL
Urine Pregnancy Test	Negative	Negative
Blood Urea Nitrogen (BUN)	10 mg/dL	6 to 24 mg/dL
Creatinine	0.76 mg/dL	Female: 0.59 to 1.04 mg/dL
Calcium	9.5 mg/dL	8.4-10.2 mg/dL

**Figure 1:**
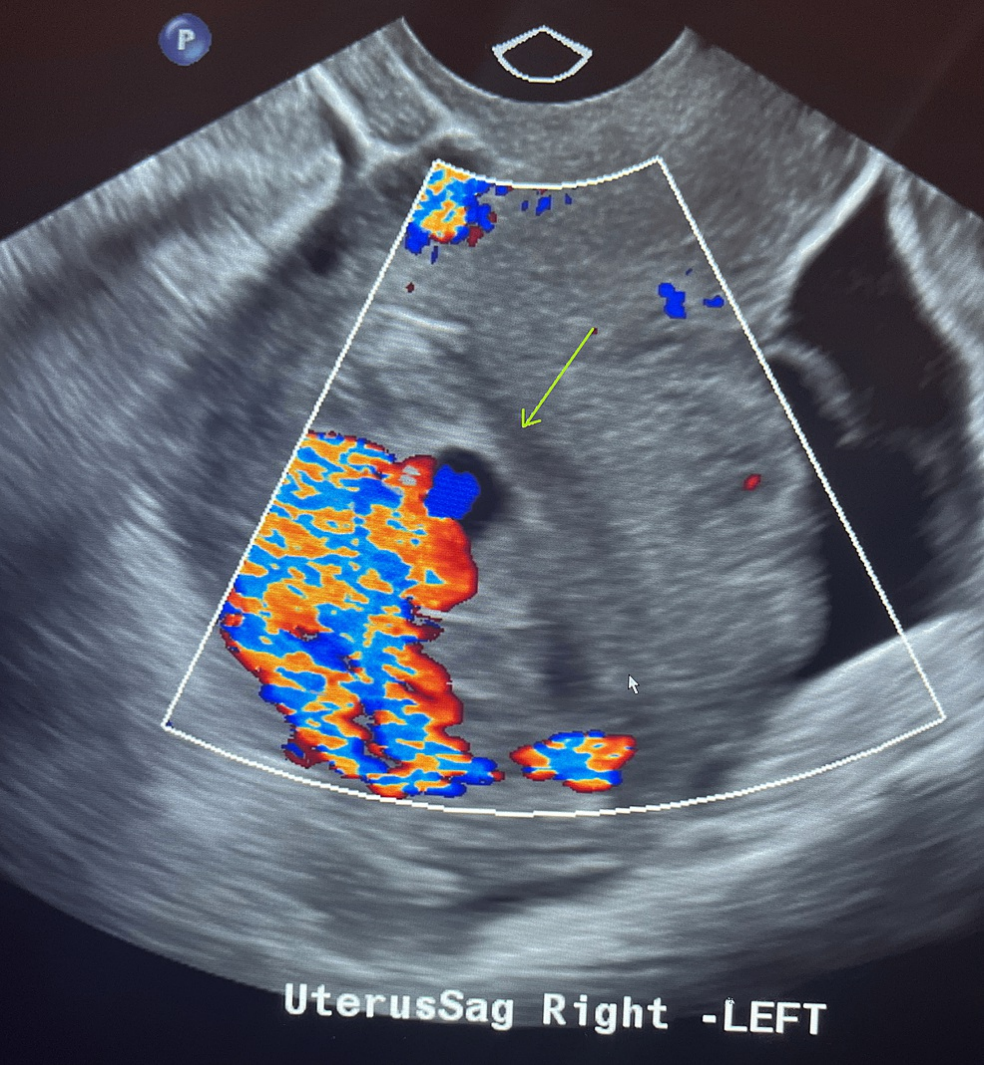
Ultrasound with Doppler. The arrow is pointing towards the endometrium, which displays vibrant abnormal flow on color Doppler.

**Figure 2:**
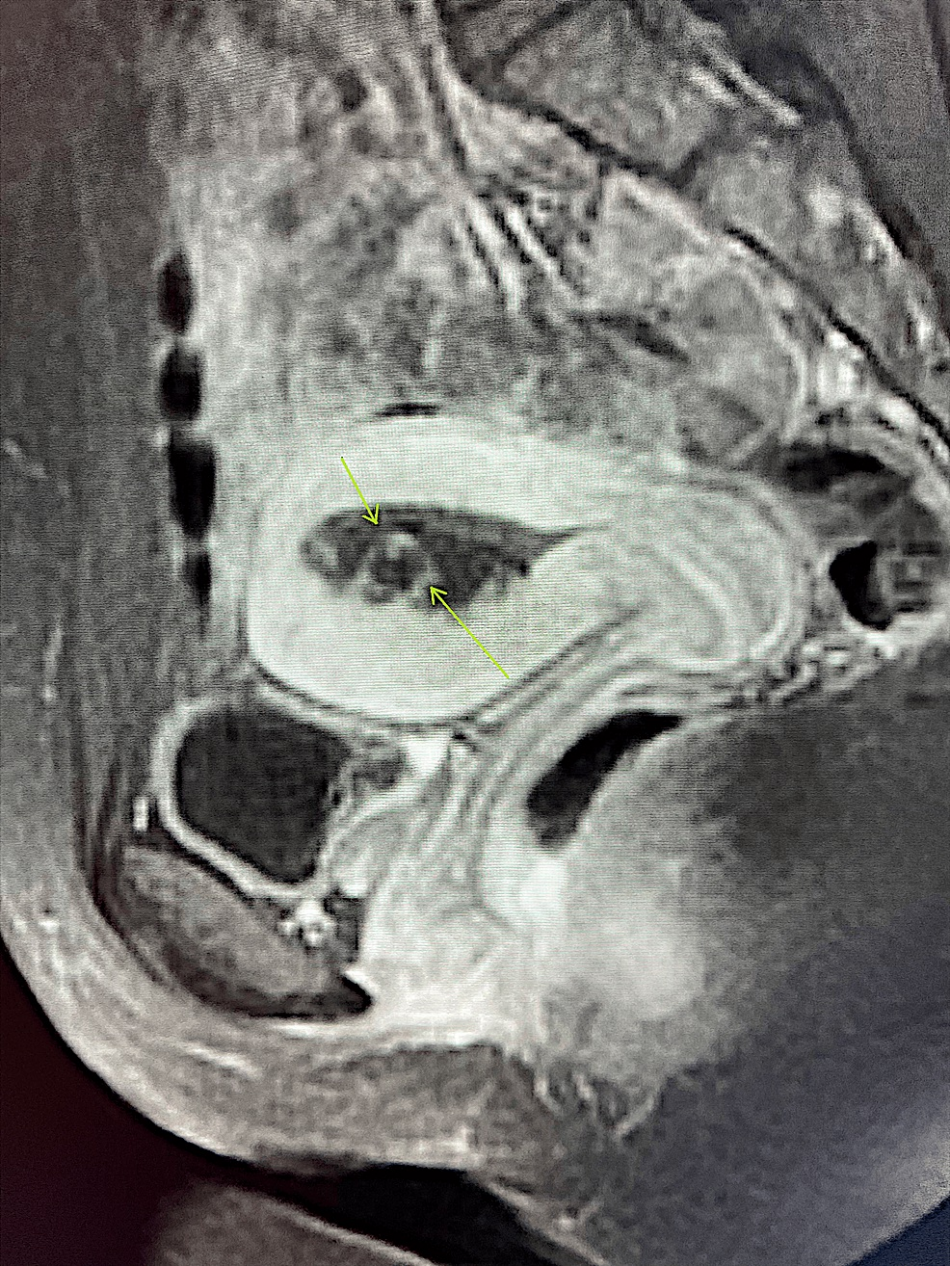
Sagittal T1 post-contrast MRI. Arrows point towards abnormal enhancement in the endometrium.

A computed tomography (CT) scan was ordered of the patient’s abdomen and pelvis. The CT demonstrated no abnormalities in the adrenals, kidneys, and other abdominal organs. Free fluid was seen in the pelvis, and the uterus was described as having a prominent endometrial cavity. The diagnostic radiologist recommended correlation with an ultrasound study. A further transvaginal ultrasound (TVUS) revealed vibrant abnormal flow on color Doppler, a finding that is concerning for an AVM (Figure 1). Obstetrics and gynecology were consulted. At this point, the patient was considered stable and discharged from the emergency department with close follow-up. Approximately two weeks later, the patient underwent a pelvic MRI revealing pathological abnormal enhancement in the endometrium (Figure 2). She reported two additional episodes of spontaneous, self-resolving, heavy vaginal bleeding at that time.

**Figure 3:**
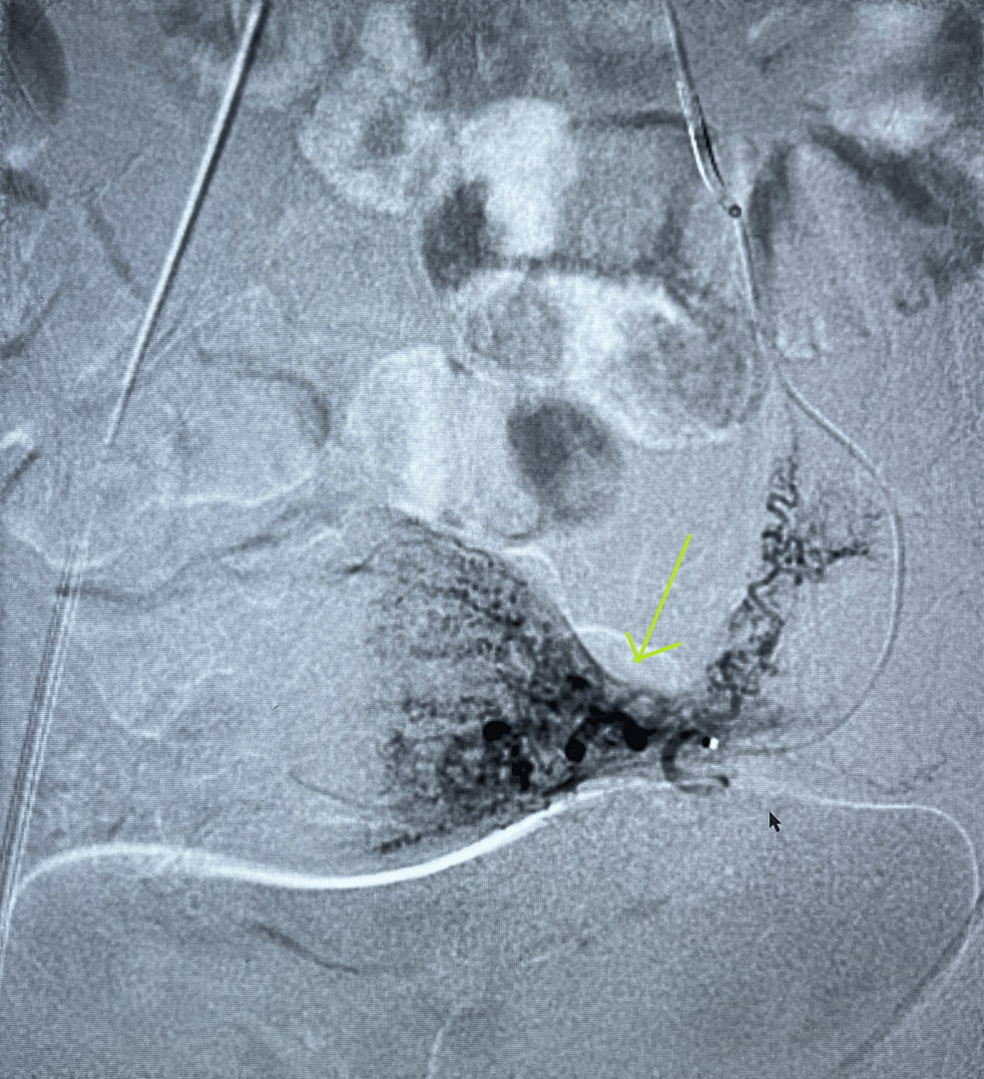
Angiographic imaging demonstrating normal left uterine artery with normal enhancement of the uterus and fallopian tube.

**Figure 4:**
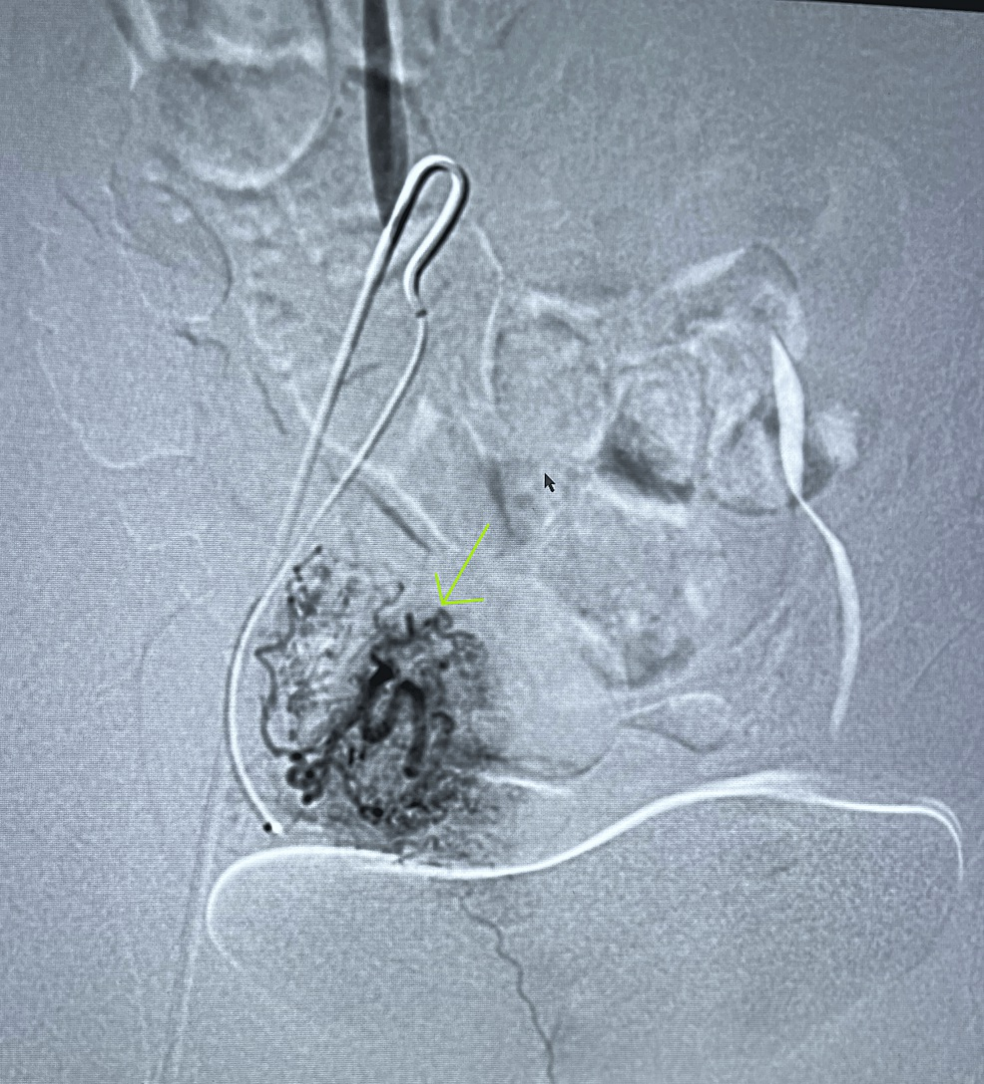
Angiographic imaging demonstrating normal right uterine artery with normal enhancement of the uterus and fallopian tube.

At this point, interventional radiology was consulted to evaluate for embolization of the suspected uterine AVM. The patient, following informed consent, opted for a uterine artery embolization. On the day of the procedure, the patient was placed in the supine position. The right groin was prepped in a sterile fashion, and lidocaine was used as a local anesthetic. The procedure was begun using real-time ultrasound guidance with a micro-puncture needle to gain access to the right femoral artery. Following initial access, a 5-French vascular sheath was inserted. A catheter was placed over the wire and used to select the left common iliac artery, where further angiography was performed. This allowed the physician to identify the uterine artery, after which the microcatheter was advanced into it. A further angiogram of the uterine artery was performed and revealed a normal appearance of the uterus and fallopian tube with no evidence of arterial venous malformation or fistula. The interventional radiologist then performed angiography from the right uterine artery, which also demonstrated a normal appearance without evidence of AVM. At this point, the case was reviewed with another interventional radiologist who agreed, based on the angiographic images, that the uterine arteries were normal and without evidence of AVM (Figures 3-4).

Finally, the case was discussed with the patient’s family who agreed with no further intervention at this point. The most likely theory for the absence of AVM on angiography was spontaneous self-resolution of the AVM, given the time frame from the initial presentation to the procedure. Follow-up with the patient revealed no further episodes of abnormal vaginal bleeding. 

## Discussion

As previously discussed, treatment varies widely based on multiple factors. These factors include hemodynamic instability at presentation, which was not a concern for our patient [1,5]. Another important factor to consider is a history of heavy bleeding. The patient had endorsed multiple episodes of significant bleeding and presented in apparent distress, although no signs of volume depletion, hemodynamic instability, or anemia were detected through physical examination or basic laboratory evaluations. Nonetheless, a history of heavy bleeding was a sufficient cause based on current literature to consider further medical or surgical management [1,5]. Discussion with the patient and her preferences led physicians to consider a minimally invasive embolization, following current literature [1,5]. The possibility of spontaneous resolution was discussed with the patient [5]. It was known that this was most likely an iatrogenic procedure-linked acquired AVM following her D&C two months prior. Current literature made it known that this type of AVM had a higher likelihood of self-resolution than congenital AVMs, although no firm number on the actual rate was consistent between studies [5,9]. Furthermore, the patient desired a more active course of management, and following informed consent wished for the procedure.

The question we must ask ourselves now is how we could have avoided this intravascular procedure which, in hindsight, was unnecessary. Although the physician was aware of the possibility of spontaneous self-resolution of the AVM, existing literature offered no guidance or actual management protocol. Due to the relatively short period elapsed between the initial visit and the actual procedure, there was no apparent utility to repeat imaging immediately before the procedure. This is logical when considering this woman had undergone multiple scans, all of which had significant costs, both for the patient and for the hospital's imaging resources [11]. The hypothesis that could be made based on this case would be to consider repeating a simple TVUS immediately prior to any kind of surgical procedure for the AVM [12]. This would avoid the radiation exposure of a CT and be much more affordable for the patient than a second MRI [11]. An ultrasound is a widely accepted method for an initial diagnosis of a uterine AVM, although further modality is usually recommended for confirmation [1,7]. In a case where the AVM had already been confirmed using additional imaging modalities, we would like to theorize that a repeat TVUS would be a good method to confirm if the AVM had spontaneously self-resolved prior to the invasive procedure. To support this affirmation, a literature review on multiple modalities and techniques of ultrasonographic imaging explains that a Doppler using specific parameters had an accuracy of greater than 85% for diagnosing uterine AVM [12]. This previous affirmation is simply a hypothesis that would require further data and cases to confirm, but this would be a good avenue for future work to prevent unnecessary procedures in women with acquired uterine AVMs [12]. Another consideration is that there is a possibility that this may have simply been enhanced myometrial vascularity instead of a true AVM, although both interventional radiologists involved in the case supported the true AVM diagnosis.

The final learning point for this case study would be to reevaluate and further discuss the criteria for medical management of a uterine AVM. There is no consensus on criteria for medical versus surgical management apart from hemodynamically stability and access to reliable follow-up for medical management [2,13]. The literature review previously mentioned did go on to explain that selection for medical management versus intravascular or surgical interventions should be an individual choice following a discussion between the patient and physician, which is effectively what was done in our case [13]. Of interest, this paper describes the four pharmaceutical classes used for medical management with the highest rate of success, which, in order would be uterotonics (100%), methotrexate (90.9%), gonadotropin-releasing hormone (GnRH) agonist (89.3%) and progestins (82.5%) [13]. The point we are trying to make is that as previously hinted in the case presentation, the patient may have unknowingly been medically treated given that she had chosen to receive a medroxyprogesterone acetate injection following miscarriage and D&C for birth control within the two months prior to presentation. While this form of progesterone was described in the literature as an effective treatment, the timing makes its effect unclear, inducing a significant confounder in this case [13]. Progestin treatment is described as effective when given following bleeding, so its usefulness when received many weeks prior is uncertain. Further data is required to understand the role that this injection had in the self-resolution, if any. Nonetheless, with hindsight this patient would have been a good candidate for medical management, demonstrating the need for further data to promote specific criteria for selection of medical versus surgical management.

## Conclusions

We hope that this uncommon case of trauma-induced uterine AVM associated with miscarriage and D&C highlights a potential consequence of uterine trauma. We also hope that this specific case highlights two major points. First, although further work on this topic and data is required to support this affirmation, we believe a pre-operative TVUS should be performed prior to any surgical intervention for uterine AVM when any significant time has passed between diagnosis and the intervention in question. This may help avoid unnecessary interventions while minimizing radiation exposure and financial burden to the patient. Additionally, we have underlined the dire need for further study and elaboration of criteria to assist physicians in selecting patients for medical versus surgical management of uterine AVMs. Finally, it would be useful to study the incidence of uterine AVMs in women choosing medroxyprogesterone injections as birth control following birth or D&C, as well as studying any variation in rates of spontaneous resolution.
